# Collaborative Indoor Access Point Localization Using Autonomous Mobile Robot Swarm

**DOI:** 10.3390/s18020407

**Published:** 2018-01-31

**Authors:** Fahed Awad, Muhammad Naserllah, Ammar Omar, Alaa Abu-Hantash, Abrar Al-Taj

**Affiliations:** Department of Network Engineering and Security, Jordan University of Science and Technology, P.O. Box 3030, Irbid 22110, Jordan; mmnaserllah12@cit.just.edu.jo (M.N.); aaomar120@cit.just.edu.jo (A.O.); amabuhantash12@cit.just.edu.jo (A.A.-H.); amaltaj12@cit.just.edu.jo (A.A.-T.)

**Keywords:** indoor localization, rogue access point, collaborative, mobile robot swarm, Received Signal Strength Indicator, Wi-Fi, simulation, experimental testing

## Abstract

Localization of access points has become an important research problem due to the wide range of applications it addresses such as dismantling critical security threats caused by rogue access points or optimizing wireless coverage of access points within a service area. Existing proposed solutions have mostly relied on theoretical hypotheses or computer simulation to demonstrate the efficiency of their methods. The techniques that rely on estimating the distance using samples of the received signal strength usually assume prior knowledge of the signal propagation characteristics of the indoor environment in hand and tend to take a relatively large number of uniformly distributed random samples. This paper presents an efficient and practical collaborative approach to detect the location of an access point in an indoor environment without any prior knowledge of the environment. The proposed approach comprises a swarm of wirelessly connected mobile robots that collaboratively and autonomously collect a relatively small number of non-uniformly distributed random samples of the access point’s received signal strength. These samples are used to efficiently and accurately estimate the location of the access point. The experimental testing verified that the proposed approach can identify the location of the access point in an accurate and efficient manner.

## 1. Introduction

Wi-Fi networks are almost everywhere nowadays, at homes, workplaces, and even in public places like malls, parks, and bus stations. Although the technology is widely spread out, it still suffers from security threats and lacks the state-of-art ways of deploying it. Rogue access points, which are unauthorized access points installed without prior authorization from network administrators for malicious purposes, are a well-known problem in Wireless Local Area Network (WLAN) security [1]. These access points can negatively influence the experience of the users and more dangerously, can potentially be used to steal their credentials. 

For indoor localizations, several different techniques have been recently proposed to provide robust and reliable localization accuracy (e.g., [2,3,4,5,6,7,8,9]). However, most of these techniques either require special hardware to estimate the distance to the transmitter or extra cost of data collection, storage resources, and/or intensive processing of complex algorithms in order to estimate the location. In Wi-Fi-based indoor localization, the most popular and cost-effective method to estimate the distance to the transmitter is via the Received Signal Strength Indicator (RSSI).

Identifying the position of a hidden Access Point (AP) usually involves laborious human work at the site by taking samples of the RSSI level at various known locations. The relationship between the collected RSSI values and the distance, d, from the AP is expressed as follows:

(1) $$RSSI(d)=RSSI(d_{0})-10n\log\left(\frac{d}{d_{0}}\right)+\chi_{\sigma }\;dB$$

where $RSSI(d_{0})$ is the received signal strength at some reference distance, $d_{0}$, *n* is the path loss exponent, which indicates how fast the received signal strength decreases with respect to distance and it depends on the environment, and $\chi_{\sigma }$ is a normally distributed random variable with zero-mean and standard deviation of σ [10]. Estimating the distance based on the RSSI is troublesome because the RSSI keeps fluctuating randomly, based on $\chi_{\sigma }$. To estimate the distance to the AP at a certain location based on (1), a sufficient number of sample RSSI’s need to be collected and averaged in order to mitigate the effect of $\chi_{\sigma }$. In addition, $RSSI(d_{0})$ and *n*, which are environment-dependent and experimentally estimated, need to be known a priori. This requires repetitive labor-intensive and time-consuming experimental testing at each location. Therefore, for practical purposes, simple, fast, and environment-independent AP location identification mechanisms are in high demand.

Several methods were proposed in the literature to identify the location of a hidden AP using RSSI in indoor environments. In [11], the RSSI is measured at several locations around a given position in order to enhance the accuracy of the associated triangulation calculations. This is repeated for a fair number of positions within the premises. The triangulation calculation may be performed after each iteration. Such triangulation calculations render a vector directed toward the AP of interest. This operation is repeated until a sufficient accuracy of the vector is reached. Even though this method can achieve an acceptable level of accuracy, the complex calculations of triangulation can quickly drain the battery of the mobile devices, which adds a considerable drawback to it since the power efficiency is of big concern nowadays. 

In [12], the relation between RSSI and distance was linearly approximated in order to estimate the location of the AP. The paper targeted the problem that the values of $RSSI(d_{0})$ and *n* may not be obtained in anonymous environments and that the exponential relationship between distance and RSSI is not solvable. Hence, the simpler linear approximation solution of the distance is proposed. However, the performance of the proposed scheme was evaluated only via simulation, yet there is a constraint that RSSI levels should be measured at least at four different positions a priori, according to the authors. 

The approach proposed in [13] uses crowdsourcing with nonlinear weighted least squares to estimate the location of the AP along with the signal propagation characteristics. The algorithm does not require a priori knowledge of the propagation parameters and it works fairly well in indoor environments. However, it requires collecting random RSSI samples within the environment in a traditional fashion, which requires intense labor and a relatively long time of data gathering. In addition, it was found that the algorithm does not perform well when the AP is close to the fringe of the environment under investigation, especially the corners [14]. 

Some large-scale AP localization systems use the Centroid and Weighted Centroid algorithms [15,16]. The Centroid algorithm determines the geometric mean of a large number of sample locations that are taken all around the AP coverage area [15]. Hence, it determines the average of all locations around the AP regardless of the signal strength at these locations. On the other hand, the Weighted Centroid algorithm uses the RSSI at each location as a weight for the coordinates by multiplying a scaled version of the RSSI by each coordinate then dividing by the sum of the scaled RSSI values [16]. Thus, more significance is given to the sample locations with stronger RSSI, which is intuitive since the stronger the RSSI, the closer the AP. However, even though the farther sample locations are given less weight, and hence have less significance in determining the AP location, they still contribute to the positioning error. It is worth mentioning that these methods are mostly used in metropolitan-scale outdoor environments. 

There are other algorithms that focus on the simplicity of the operation, without sacrificing the accuracy. The Where is My Access Point (WiMAP) algorithm introduced a simple, yet efficient, solution to identify the location of an AP [17]. The WiMAP algorithm proposed the use of the basic Centroid algorithm in a simpler filtering process than the Weighted Centroid algorithm. It consists of three simple phases: collecting samples, filtering the collected samples, and estimating the AP location. In the sample-collecting phase, WiMAP relies on collecting a uniformly distributed set of RSSI samples at known locations without any prior knowledge of the AP configuration (e.g., the transmit power) or the signal propagation characteristics of the specific environment (e.g., $RSSI(d_{0})$ or *n*). In the filtering phase, the collected samples are sorted and filtered based on the RSSI level. In the location estimation phase, the centroid algorithm is applied to the samples’ locations, except that the geometric mean is calculated only for the selected fraction of the collected and sorted sample points. The AP location is estimated as the centroid of these samples’ locations. The experimental testing demonstrated high precision in estimating the location of the AP, especially when it is not very close to the fringe of the area under investigation. 

In [18], we introduced an enhanced and more efficient version of WiMAP, called Dynamic WiMAP (DWiMAP) and evaluated its performance, compared to WiMAP, using an autonomous mobile robot in an indoor environment. Unlike WiMAP, DWiMAP dynamically and selectively collects a relatively small number of the most relevant RSSI samples in a non-uniform manner. Based on the received RSSI values, DWiMAP automatically divides the area into sectors in the form of rings around the current “Center of Interest” (the location, where the highest RSSI has been received so far), which is where the AP is at (or at least is close to). Thus, the sample density varies per sector, depending on how far or how close the sector is to the AP. The sample density increases as the sector gets closer to the center of interest and decreases as the sector is farther away from it. By doing this, the total number of samples is decreased, the redundant or irrelevant sample locations are avoided, and the time needed to complete the area scanning is shortened. This is done without affecting the accuracy, since low RSSI values have relatively low impact on estimating the position of the AP [17]. Comparative analysis demonstrated that DWiMAP outperforms WiMAP in both time and number of samples, without sacrificing the localization accuracy, which makes it appealing for real-life practice.

In this paper, we introduce an efficient and practical collaborative approach to identify the location of an AP in an indoor environment using a swarm of wirelessly connected mobile robots. We further introduce distributed versions of WiMAP and DWiMAP, which are deployed within the robots for experimental testing and comparative performance evaluation and analysis. The mobile robots collaboratively and autonomously collect a set of non-redundant samples of the AP’s RSSI level according to the corresponding algorithm. These samples are used to efficiently and accurately estimate the location of the access point. The experimental testing verified that the proposed approach can identify the location of the AP in an accurate and efficient manner.

The significance of the proposed approach is in providing an efficient, fast, accurate, and low-cost alternative to human-centric approaches. In RSSI-based positioning strategies, taking a precise RSSI measurement and an associated location information, whether for off-line fingerprinting or on-line location estimation, is very critical for achieving robust location estimation. The process of collecting RSSI measurements at specific locations, especially in large-scale environments, is very labor-intensive and time-consuming. When such a task is assigned to a human being, the chance of obtaining a reliable set of RSSI measurements is low due to the imperfect nature of humans; humans tend to get tired and bored and hence make mistakes after repeating the same routine of movements a number of times, carrying a mobile device, staring at it, etc. In addition, humans cannot reach small and narrow places, which may create holes in the RSSI mapping, and the device can easily become shadowed by the human body, causing inaccurate RSSI measurements. Therefore, the human factor makes it slow, imprecise, inconsistent, and unreliable, especially if the process is to be repeated regularly (e.g., searching for, localizing, and arresting rogue access points within a service area). The experimental testing demonstrated that a mobile robot swarm can perform such tasks in a very precise, fast, low-cost, and robust manner.

## 2. Methods 

A Mobile Robot Swarm is a new strategy that describes the coordination of multi-robot systems collaboratively accomplishing a specific task, or set of tasks, in an efficient and autonomous manner, within an unknown environment [19]. One of the main benefits of using multi-robot systems is multi-tasking, where a number of mobile robots perform a set of tasks simultaneously in a coordinated and optimized manner. Therefore, task allocation plays an important role in multi-robot systems [20]. This is because the allocation of tasks can significantly affect the overall performance of the system in terms of time, energy, and any other relevant performance metric used. 

One important research question is how robot swarms can make collective decisions, a need that arises in many applications of swarm robotic systems. Collective decision-making in robot swarms typically requires some form of consensus among the individual robots of the swarm. Existing methods typically rely on measuring the quality of the available options, followed by some form of explicit negotiation or consensus finding (e.g., [21]).

Nevertheless, along with multi-tasking, mobile robots have to employ some localization and navigation mechanisms in order to reach the locations, where the tasks assigned to them are to be executed. Each robot should have the capability to identify its own location, as well as the locations of other robots in the swarm, in order to be able to move towards the target location, while avoiding obstacles and staying within a safe distance from other robots.

Since the robots may move to areas of far distance, it is important to have a pervasive networking environment for communications among the robots, administrators, and mobile users. In [22], the authors proposed an architecture called robot swarm communication networks, where the robots are clustered to one or multiple swarms. Each swarm can be monitored and controlled by a central server through a wireless mesh backbone, as well as the Internet. Within each swarm, a self-organizing mobile ad hoc network is formed such that all robots are connected to all other robots despite the movements. Meanwhile, mobile users can also monitor swarms through mobile devices.

In this work, we introduce a collaborative multi-robot system using a commercially available indoor robot platform. Each robot has proximity sensors for obstacle avoidance and Wi-Fi radio for communication. The robot swarm is designed to autonomously form a mobile ad hoc network and keep track of all reachable robots in the swarm and their current locations, even if they are not within the communication range of each other. To demonstrate the effectiveness of the system in practical applications, distributed versions of the WiMAP and DWiMAP AP localization algorithms were implemented in a system of four mobile robots, forming a swarm, which was shown to collaboratively and efficiently identify the location of the AP in an indoor environment.

### 2.1. Robot Platform and Testing Environment

The robot platform used in this work is the Khepera III (Kh3) [23], which is operated by the standard embedded Linux Operating System. Kh3 has two wheels, where each wheel is coupled with a DC motor to allow the robot to move and turn in different directions in a smooth way. It also includes a standard Compact Flash extension card slot that supports Wi-Fi and Bluetooth wireless networking technologies, which allows the platform to communicate easily with an operating computer system for programming and monitoring purposes. It also allows a group of robots to form a mobile ad hoc network.

Kh3 has a body-mounted array of 11 infrared proximity sensors, as well as 5 ultrasonic range sensors. Therefore, Kh3 can detect obstacles, whether they are very close (i.e., less than 20 cm) using the infrared sensors, or far (i.e., between 20 cm and 4 m) using the ultrasonic sensors. Thus, it can also be programmed to avoid such obstacles, whether stationary or moving, in a dynamic and seamless fashion, as discussed later.

**Figure 1:**
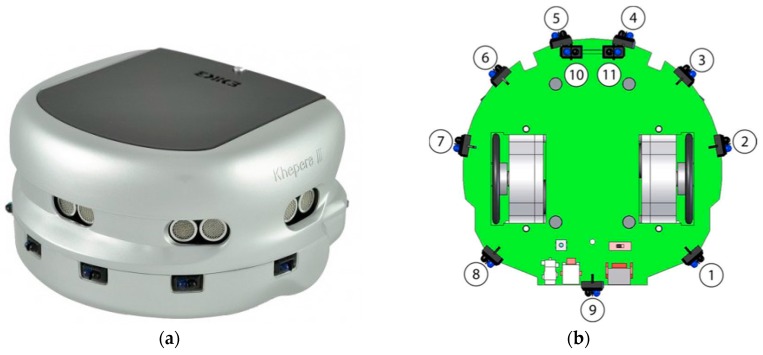
The mobile robot platform [23]: (**a**) Khepera III robot; (**b**) a bottom view of the robot showing the locations of the infrared sensor.

Figure 1a shows Kh3 robot platform with the ultrasonic sensors along with the front infrared sensors and Figure 1b shows a bottom view of the robot body with the positions of the infrared sensors and the positions of the wheels. Note that Kh3 has 2 infrared sensors attached to the front bottom of the body to prevent the robot from falling off an edge.

**Figure 2:**
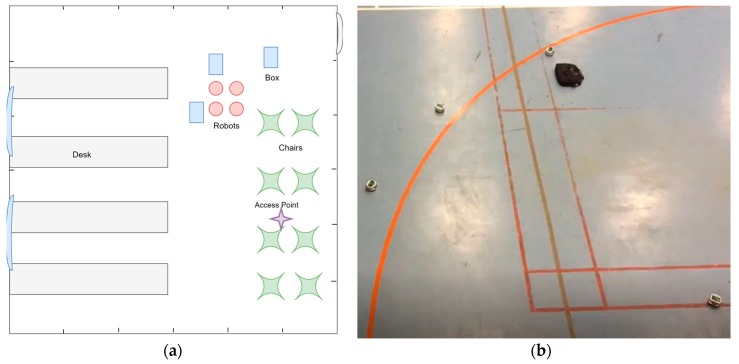
Samples of the environments used for experimental testing: (**a**) a sketch of a heavily obstructed small office space (training/meeting room); (**b**) a snapshot of a lightly obstructed large open space (gymnasium).

During the course of this study, the environments used in performing the experimental testing were all obstructed indoor environments. Some were relatively small rooms such as an office or a training room. Others were relatively large and open spaces such as gymnasiums or corporate-type office spaces. Since locating an AP in a small room is not very challenging, most of the experimental testing was performed in large open spaces. Within these spaces, the robots were programmed to navigate through three area sizes: 5 m × 5 m, 8 m × 8 m, and 10 m × 0 m. These spaces include naturally existing obstacles such as chairs, desks, basketball hoops, volleyball nets, and humans moving around in addition to intentionally placing small obstacles within the testing area such as boxes, etc. Figure 2 shows two sample indoor environments used for experimental testing.

### 2.2. Robot Swarm Communication System

In order to facilitate the communication and status update and information exchange among the robots in the swarm, a Reliable Communication System (RCS) was implemented in each robot in an ad hoc distributed fashion. RCS provides each robot with two separate communication channels:

1. A control channel, which allows the robot to periodically advertise its own existence and realize the existence of other peer robots in the environment. To achieve that, two types of packets are exchanged among nodes via broadcasting:
  a) Beacon packets: to periodically advertise that the robot is alive.
  b) List packets: to advertise any update on the alive robot list in the network. Each robot builds a list of IP addresses of the active robots and updates it periodically based on “Bellman-Ford” algorithm [24].
  Every change in the status of a robot in the network is updated. For example, if a robot leaves the area (i.e., becomes out of reach as no beacon packet are received from it for a predefined period of time) or a new robot joins, the list is updated by adding a new entry for the joining robot or removing the entry of the robot that left.
2. A data channel, which is used for exchanging and sharing different kinds of information with the rest of the robots in order to perform a specific task. For example, to collaboratively detect the sizes and shapes of obstacles in the area, every time a robot detects an obstacle, it shares its location and the readings and directions of its proximity sensors with all other robots. This results in discovering the approximate shape of the obstacle since each robot is likely to approach it from a different direction. In addition, each robot can exchange and keep an up-to-date list of different real-time parameters about the other robots for coordination purposes, such as battery level, current location, direction of movement, etc., which can be used to coordinate and optimize the execution of some required tasks.

Note that the control channel provides each robot with an updated list of live active robots in the environment in real-time. On the other hand, the data channel provides each robot with the updated list of parameters and discovered information by other robots in real-time such as the locations of obstacles, etc. Thus, it does not matter how many robots participate in the process, when a new robot joins or when an existing robot leaves. The process in each robot keeps running until the corresponding algorithm exits normally. That is, whether new robots join the swarm during execution or other robots leave, if they lose communication with the swarm, or if they run out of battery, the execution time may change accordingly. The localization accuracy is independent of the number of robots involved or when each robot joins or leaves the swarm. In fact, during the experimental testing, it happened that two robots ran out of battery. The rest of the robots continued their tasks without any problem and covered for the departing robots. This makes the robot swarm system highly robust.

### 2.3. Robot Navigation and Localization

Each robot was equipped with a navigation and localization mechanism that is responsible for guiding the robot to the location of the next task to be executed, while keeping track of its own current location and avoiding obstacles in the way. 

For obstacle avoidance, a simple and efficient algorithm was designed and implemented in each robot based on both Braitenberg’s concept in vehicles [25] and the virtual force field mechanism [26]. A Braitenberg vehicle is an agent that can autonomously move around based on its sensor inputs. It has primitive sensors that measure some stimulus at a point and wheels (each driven by its own motor) that function as actuators or effectors. In the simplest configuration, a sensor is directly connected to an effector so that a sensed signal immediately produces the movement of the wheel. Thus, the motion of the vehicle is directly controlled by sensors, yet it results in a behavior that may appear complex or even intelligent. The Braitenberg concept is mostly suitable when there is a separate motor to each wheel on the robot, which is the case in robot platform used.

In the proposed obstacle avoidance algorithm, the Braitenburg concept was applied to both the ultrasonic sensor set and the infrared sensor set. This allows the robot to avoid static objects that are 20 cm to 4 m away at an early stage using the ultrasonic sensors and, at the same time, avoid mobile obstacles that may suddenly appear at close proximity to the robot using the infrared sensors. Thus, each sensor (whether an ultrasonic or an infrared) is assigned a specific weight for each motor and the sensor readings are weighted accordingly. The combined weighted sensor readings of each motor control the rotation speed of the corresponding wheel as follows:

(2) $$S_{L}=S_{L}+\overset{N}{\underset{i=0}{\sum }}W_{L}\left(i\right)\times R\left(i\right)$$

(3) $$S_{R}=S_{R}+\overset{N}{\underset{i=0}{\sum }}W_{R}\left(i\right)\times R\left(i\right)$$

where $S_{L}$ and $S_{R}$ are the left and right motor speeds, respectively, *N* is the number of sensors, $W_{L}\left(i\right)$ and $W_{R}\left(i\right)$ are the weights of sensor *i* corresponding to left and right motors, respectively, and R(i) is the reading of sensor *i*.

To determine the most suitable weights for each sensor, random weights were first assigned and then experimentally adjusted a number of times until smooth movement around the obstacles were achieved. Table 1 and Table 2 list the sensor weights for the ultrasonic and infrared sensor used in the experiments, respectively.

To allow a robot to maneuver freely around obstacles towards the target location, in addition to reducing the amount of computations, far away obstacles are ignored. That is, if the sensor reading indicates that the distance to the obstacle is larger than a certain threshold, the reading is set to zero (e.g., for ultrasound sensors, the threshold distance was set to 80 cm). 

**Figure 3:**
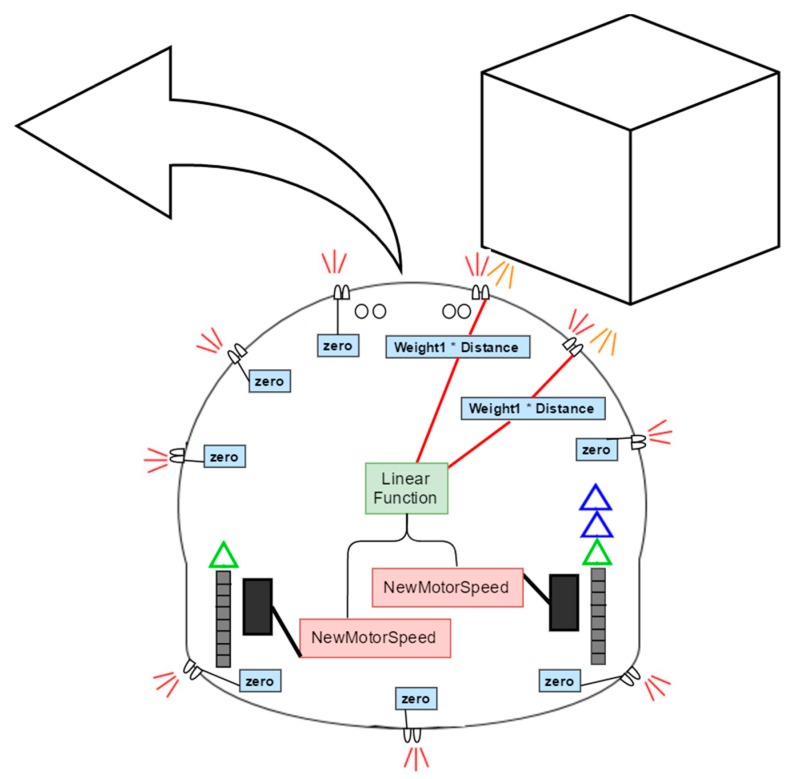
The obstacle avoidance mechanism as implemented in the mobile robot platform.

Figure 3 illustrates the obstacle avoidance mechanism and how the sensor readings are used to turn the robot away from obstacles in the correct direction through the best obstacle-free direction. Note that the results for the back and left sensors are zeroes since no obstacle is detected within the threshold distance, which keeps the rotational speed of the left motor nearly the same. On the other hand, the non-zero weighted readings of the of right sensors increases the rotational speed of the right motor, causing the robot to smoothly rotate counterclockwise around its center, which results in a smooth turn left around the obstacle.

For robot localization, a technique called Odometry was used [27], which is based on tracking the rotation and direction of the wheels. Odometry works well when the wheels are equipped with high precision encodes that measure the rotation of the wheels accurately, which is the case for the robot platform used. Usually, Odometry does not work well in very long paths due to accumulative errors. The reason it is used in this research is the lack of a better alternative for large-scale indoor localization or location identification system. In the results section, we show via experimental testing, that the mobile robot platform used has a relatively accurate Odometry system.

For a two-wheeled robot, within a short time interval, ∆t, the velocities of two wheels are relatively constant and the robot’s forward and rotational velocities can also be considered constant. Hence, the position update from $P_{1}=\left(x_{1},\;y_{1}\right)$ to $P_{2}=\left(x_{2},\;y_{2}\right)$ can be expressed as follows:

(4) $$x_{2}=x_{1}+d\cos\theta$$

(5) $$y_{2}=y_{1}+d\sin\theta$$

where *d* is the average distance traveled by the robot and *θ* is the direction of movement, which can be expressed as:

(6) $$d=\frac{d_{L}+d_{R}}{2}$$

(7) $$\theta =\frac{d_{L}-d_{R}}{L}$$

where $d_{R}$ is the distance travelled by the right wheel, $d_{L}$ is the distance travelled by the left wheel, and *L* is the distance between the wheels.

It is worth noting that when a robot makes a turn, its wheels do not actually turn, it is rather that one wheel moves faster than the other does, causing the robot to turn, and the angle of such a turn is based on the speed difference between the two wheels.

### 2.4. Distributed WiMAP and DWiMAP for the Multi-Robot System

To make WiMAP and DWiMAP work in a collaborative fashion using a multi-robot system, a distributed version of each protocol was devised and implemented within each robot. The core difference between the standalone version and the distributed version of either algorithm relies on sharing the measurements data among the robots via RCS in real-time. Each robot adapts its decisions and movements based on the shared information. 

To make the navigation within an obstructed environment easier, the area under investigation is virtually divided into a grid of 10 cm × 10 cm cells. Each cell is assigned one of three possible values based on whether it has been discovered by one of the robots or not: 0, 1, 2, and −1, to indicate “unvisited”, “visited”, “covered” (i.e., covered by a previously collected RSSI sample), and “unreachable” (i.e., occupied by an obstacles), respectively. Initially, all cells were assigned 0 s. As the robots navigate through the environment, all cells that are within range of the body-mounted range sensors are marked appropriately. 

To guarantee an efficient coverage of the area of interest with appropriate RSSI samples, while keeping the distance travelled by the robots at minimum (hence saving time and energy), a simple distributed random search mechanism for the next valid sample location was developed. Despite their simplicity, techniques for random exploration of unknown environments demonstrated high efficiency in practice [28]. The efficiency of random search techniques was theoretically addressed in the literature, as in [29]. In this research, the robot swarm is not required to explore the whole area, as long as the RSSI sample density requirement is satisfied, but rather to swiftly collect a sufficient number of RSSI samples at random locations within an unknown environment based on an appropriate sample density. This makes the random search strategy appropriate for this research.

In the proposed random search mechanism, in order to avoid too close by or too far apart samples, a robot selects a new location to move to in random fashion within a lower and an upper threshold distances, $D_{L}$ and $D_{U}$, respectively from the current location of the robot $\left(x_{c},\;y_{c}\right)$. Very close-by samples can be redundant, increasing the overall operational overhead, whereas too far apart samples can be insufficient, which degrades the localization accuracy. Therefore, when a sample is taken within a cell, all cells around it within the initial $D_{L}$ are marked as “covered”. Otherwise, it is marked as “uncovered”. The mechanism of the random search for samples works as follows:

1. Randomly select a new location $\left(x_{n},\;y_{n}\right)$ such that the Euclidean distance from $\left(x_{c},\;y_{c}\right)$ is between $D_{L}$ and $D_{U}$. Thus:
  (8) $$D_{L}<\sqrt{{\left(x_{n}-x_{c}\right)}^{2}+{\left(y_{n}-y_{c}\right)}^{2}}\leq D_{U}$$
2. If the cell of the new location is not marked as “unreachable” or “covered”, move to the new location.
3. If the new location is found to be “unreachable”, select an alternative close by cell that meets the above criteria.
4. If no more eligible cells are found, the search area is widened such that $D_{L}=D_{U}$ and $D_{U}=D_{U}+c$ and the process is repeated until the whole area is covered, where c is a constant distance.
5. The only difference in the search process between WiMAP and DWiMAP is that in WiMAP, the initial values of $D_{L}$ and $D_{U}$ are always the same, whereas in DWiMAP, the initial values of $D_{L}$ and $D_{U}$ are inversely proportional to the RSSI level at $\left(x_{c},\;y_{c}\right)$. The reason is that WiMAP requires the samples to be uniformly distributed in the area of interest, whereas in DWiMAP, the closer to the AP location, the higher the sample intensity should be.

The pseudo code of the random search process is shown in Algorithm 1.

**Algorithm 1** FindNextSampleLocation1: **function** FindNextSampleLocation (*Grid,Location, MaxSearchingRange,*
$D_{U}$, $D_{L}$, *C*)2:  *UpperSearchingRange* ← $D_{U}$3:  *LowerSearchingRange* ← $D_{L}$4:   **while**
*UpperSearchingRange* ≤ *MaxSearchingRange*
**do**5:     **while**
*UncoveredCellsExist*
**do**6:      *CandidateLocation* ← *Random* ∈ [*UpperSearchingRange, LowerSearchingRange*]7:        **if**
*Cell(CandidateLocation) is Uncovered*
**AND NOT**
*Unreachable*
**then**8:          **return**
*CandidateLoction*9:        **end if**10:     **end while**11:     *LowerSearchingRange* ← *UpperSearchingRange**12:     UpperSearchingRange* ← *UpperSearchingRange + C*13:   **end while**14:   **return**
*NULL*15: **end function**

While navigating through the area under investigation collecting RSSI samples, based on the random search process, the robots exchange their findings and update their databases accordingly in real-time using RCS. Thus, all robots in the swarm keep an up-to-date map of the environment, including the status of each cell in the environment grid, the locations of the collected sample points, and the corresponding average RSSI level. As soon as all cells in the grid are covered, the robots execute the corresponding AP localization algorithm to estimate the location of AP.

The pseudo codes of the distributed versions of WiMAP and DWiMAP are shown in Algorithms 2 and 3, respectively.

**Algorithm 2** Distributed WiMAP1: **procedure**
*WiMAP* (*Grid,Location,*
$D_{U}$, $D_{L}$, *C*)2:  **while**
*true*
**do**3:   *NewLocation* ← *FindNextSampleLocation (Grid,Location, $D_{U}$, $D_{L}$, C)*4:  **if**
*NewLocation* = *NULL*
**then**5:      **break**6:  **end if**7:  *Move toward NewLocation*8:  **while** NOT *reached NewLocation*
**do**9:      *Update Grid (VisitedAreas)*10:    *Read SensorsValues and Compute ObstacleLocation*11:    *Update Grid (ObstacleLocation)*12:    *Check ReceivePackets (SampleLocation, SampleRSSI)*13:     **for**
*each ReceivePackets*14:       *Insert SampleLocation and SampleRSSI into WiMAPList*15:   *Update Grid (CoveredAreas, initial $D_{L}$)*16:     **end for**17:  **end while**18:  *RSSIval* ← *GETRSSI*19:  *Insert Location and RSSIval into WiMAPList*20:  *Update Grid (CoveredAreas, initial $D_{L}$)*21:  *Send packet (Location, RSSIval) as Broadcast*22:  **end while**23: *Sort WiMAP List*24: *BestList* ← *BestNPercent of WiMAP Samles*25: *EstimatedAP* ← *Centroid (BestList (Location))*26: **end procedure**

**Algorithm 3** Distributed DWiMAP1: **procedure**
*DWiMAP (Grid,Location, $D_{U}$, $D_{L}$, C)*2:   **while**
*true*
**do**3:    *NewLocation* ← *FindNextSampleLocation*
*(Grid,Location, $D_{U}$, $D_{L}$, C)*4:  **if**
*NewLocation* = *NULL*
**then**5:      **break**6:  **end if**7:  *Move toward NewLocation*8:  **while**
*NOT reached NewLocation*
**do**9:      *Update Grid (VisitedAreas)*10:    *Read SensorsValues and Compute ObstacleLocation*11:    *Update Grid (ObstacleLocation)*12:    *Check ReceivePackets (SampleLocation, SampleRSSI)*13:    **for**
*each ReceivePackets*14:      *Insert SampleLocation and SampleRSSI into WiMAPList*15:   $D_{L}$ ← *DWiMAP CoveringScale(SampleRSSI)*16:   $D_{U}$ ← $D_{U}$ + *C*17:   *Update Grid (CoveredAreas, $D_{L}$)*18:     **end for**19:  **end while**20:  *RSSIval* ← *GETRSSI*21:  *Insert Location and RSSIval into WiMAPList*22:  $D_{L}$ ← *DWiMAP CoveringScale(SampleRSSI)*23:  $D_{U}$ ← $D_{U}$ + *C*24:  *Update Grid (CoveredAreas, $D_{L}$)*25:  *Send packet(Location, RSSIval) as Broadcast*26:  **end while**27: *Sort WiMAP List*28: *BestList* ← *BestNPercent of WiMAP Samles*29: *EstimatedAP* ← *Centroid (BestList (Location))*30: **end procedure**

## 3. Results

To evaluate the performance of using a collaborative mobile robot swarm in speeding up the detection of the AP location, the distribute versions of WiMAP and DWiMAP were implemented within the multi-robot system, one at a time, and were tested experimentally. Each test was repeated a few times and the averages of the trials are reported in this section. Nevertheless, since the robot platform performs Odometry-based self-localization using the body-mounted wheel rotation sensors, before performing the experimental testing, the robustness and accuracy of the robot self-localization had to be validated using a precise visual tracking system.

In addition to the experimental testing, a simulation environment that mimics the behavior of the multi-robot system, as well as RCS, was implemented, including the random search process for the new sample location and the distributed versions of WiMAP and DWiMAP. One reason to build the simulation environment is to study large-scale environments and the impact of different parameters without having to always perform the time- and labor-intense experimental testing. This also allows for more accurate insight into the system by running enough trials for each scenario in order to neutralize the impact of randomness in the search process.

### 3.1. Validation of the Robot Self-Localization System

To validate the accuracy of the body-mounted robot self-localization system, the VICON Motion Capture System [30] was used. The VICON system is designed to visually track the moving objects in three-dimensional small-scale room-size space using six cameras and special markers placed on the objects. Motion capture systems, such as VICON, may not be practically used in large-scale environments due to their high-cost, complexity, and strict installation requirements. It is rather used for verification and prove-of-concept purposes [31]. For example, VICON-equipped arenas are heavily used in the robotics fields, especially quad-rotor Unmanned Arial Vehicles (UAV), for verifying positioning and obstacle avoidance algorithms or real-time control mechanisms given the actual positions provided by the motion capture systems [32,33,34,35].

**Figure 4:**
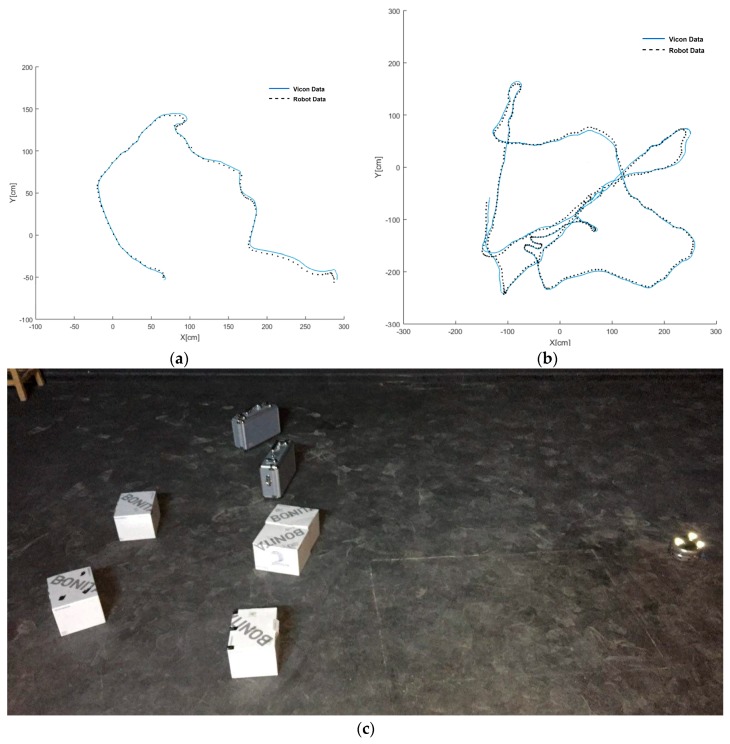
Validating the accuracy and robustness of the implemented robot self-localization system: (**a**) a short and simple navigation path; (**b**) a long and complicated navigation path; (**c**) a snapshot of the testing environment under the VICON system.

To measure the accuracy and robustness of the self-localization system, the robots were set to navigate freely and turn around obstacles within an obstructed environment under the 3 m × 3 m VICON-equipped arena in a manner similar to that of the random search process. The locations of the robots were periodically measured in real-time by both systems and results were registered and compared. The Root-Mean-Square average of the drift was less than 5 cm, which is a very decent accuracy of a mechanical system based localization. Figure 4 shows two graphical samples of the testing results with the VICON system and a snapshot of the testing environment.

### 3.2. Experimental Results

In the experimental testing, for each testing scenario, each robot was given the dimensions of the testing environment, its starting location, its starting direction, the localization algorithm (i.e., WiMAP or DWiMAP) and the upper and lower distance thresholds. Each test scenario was performed using 1, 2, 3, and 4 robots and each was repeated 3 times for a total of 24 experimental testing trials. The testing was performed close to one corner of a large open space during different days and times and while people were sporadically around whether doing their normal daily tasks or watching the running tests closely, but without interfering with them. Hence, different tests were not necessarily performed at the same exact location or under the same dynamics of the surrounding environment. This heterogeneity in the experimental testing may not produce very consistent results (i.e., the variance among different tests may be significant at times). Nonetheless, it should contribute to the robustness and reliability of the proposed methodology.

In order to mitigate the impact of variation in the RSSI (i.e., $\chi_{\sigma }$) in (1), as soon as the robot arrives at the new location where a new RSSI sample is to be collected, it collects at least 10 RSSI samples and registers their mean before moving on with the process.

For all experimental tests, $D_{L}=0.6\;m$, $D_{U}=1.0\;m$, and the location of AP along with the initial locations of the robots were randomly selected within the testing environment. The performance metrics used here are the average collected number of samples, AP localization error, and elapsed time, which is the time it took to complete the experiment. Note that, for the multi-robot scenarios, the elapsed time is what it takes the last robot to finish (i.e., the robot to cover the last cell).

**Figure 5:**
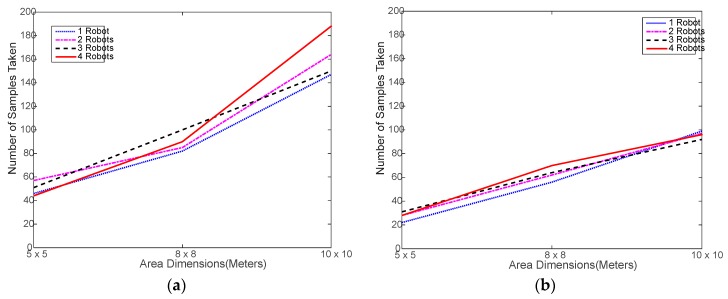
Number of samples collected in the experimental testing: (**a**) using distributed WiMAP algorithm; (**b**) using distributed DWiMAP.

Figure 5 shows that the total number of RSSI samples taken by either algorithm does not change much regardless of the number of robots used, which is a clear sign of a successful collaboration among the robots. There are some slight differences among the curves of the same algorithm, which is due to the heterogeneity of the test experiment, as discussed above. These differences are more obvious in WiMAP as it naturally collects almost double the samples of DWiMAP most of the time.

**Figure 6:**
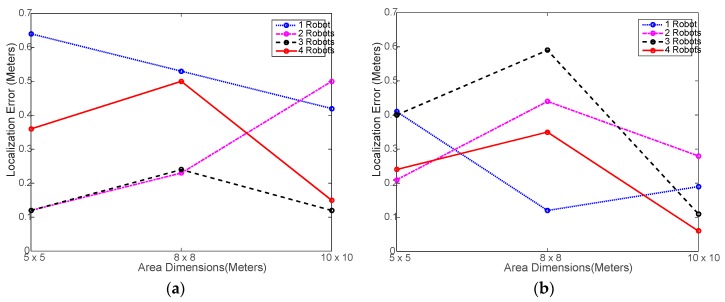
Localization error achieved in the experimental testing: (**a**) using distributed WiMAP algorithm; (**b**) using distributed DWiMAP.

Figure 6 shows that the AP localization error did not exceed 0.65 m in all case despite the high fluctuation caused by heterogeneity and small number of experiment trials. This indicates that the using the multi-robot system does not have negative impact on the localization performance whatsoever.

**Figure 7:**
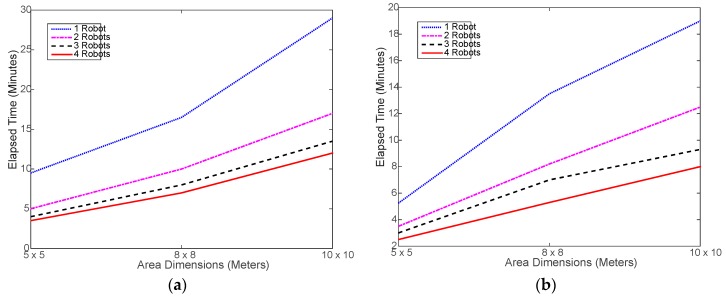
Elapsed time of running the experimental navigation and collecting required RSSI samples: (**a**) using distributed WiMAP algorithm; (**b**) using distributed DWiMAP.

**Figure 8:**
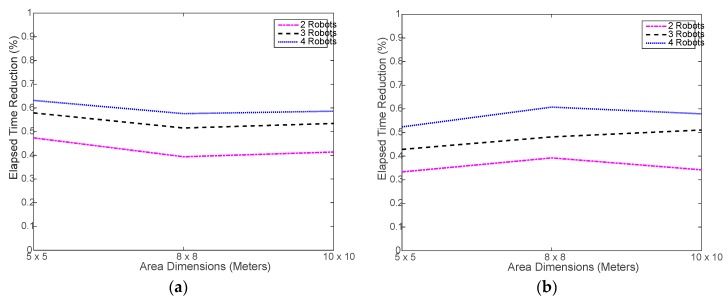
Elapsed time reduction in experimental testing: (**a**) using distributed WiMAP algorithm; (**b**) using distributed DWiMAP.

Verily, the most important advantage of using a multi-robot system is saving time via collaboration and parallel processing. Figure 7 shows that, indeed, using the robot swarm considerably reduced the time needed to execute the tasks. Ideally speaking, using 2, 3, and 4 robots should reduce the time taken by 1 robot by 50%, 67%, and 75%, respectively. However, in realty, this may not be easily achievable due to the randomness in selecting the next location to move to and the associated random obstacles in the way. Figure 8 shows the actual percent time reductions achieved experimentally, which were about 15% to 25% below the ideal reduction limits. This is mostly caused by the random nature of search for the next sample location. It may happen that the robots end up navigating in an inefficient way, as the search area get larger. During the testing, it was noticed that, in most cases, the robots do complete at the same time as some robots finish and stop earlier than the rest of the robots. This might be natural since each robot makes individual decisions during the random search process, which causes some robots to do more work. This problem is investigated in a later part of this section.

### 3.3. Simulation Results

Experimental testing requires intensive labor and time to perform large-scale experiments. In addition, each experiment has to be repeated a sufficient number of times before any conclusion can be drawn out of it. Therefore, a simulation testbed was implemented using MATLAB software platform [36]. In order to verify that the simulation testbed can precisely simulate the experimental environment, all experimental testing scenarios were mimicked using the simulation testbed for comparison purposes. To this end, the exact values of all parameters used in the experimental testing, such as the area dimensions, the random search distance thresholds, and the coverage area, along with the mechanical parameters of the robot platform, such as the average robot speed and detection ranges of the infrared and ultrasonic sensors, were used in the simulation testbed. Each experiment was repeated 50 times, each with a different random generator seed. The reported results reflect the averages of each set of 50 trials.

**Figure 9:**
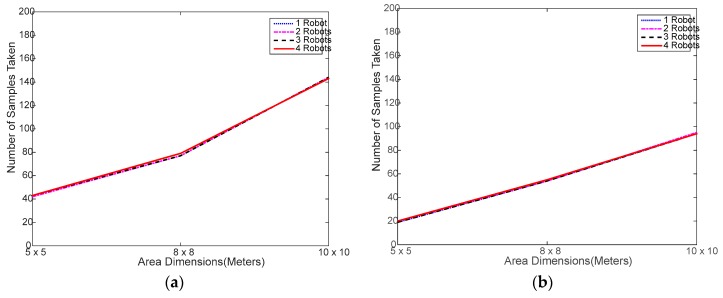
Number of samples collected in the simulation testing: (**a**) using distributed WiMAP algorithm; (**b**) using distributed DWiMAP.

Figure 9 shows that the number of samples obtained via simulation are almost identical, regardless of the number of robots used. As far as the comparison with experimental testing as in Figure 5, it is obvious that the number of samples varies with the area size in a very similar manner. This is a good indication that the simulated random search process is correctly implemented.

**Figure 10:**
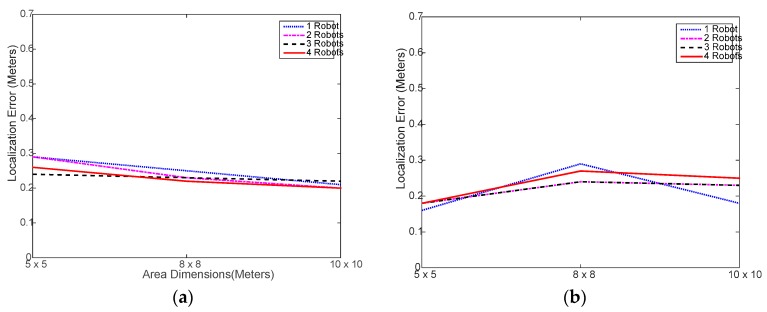
Localization error achieved in the simulation testing: (**a**) using distributed WiMAP algorithm; (**b**) using distributed DWiMAP algorithm.

Figure 10 shows again that using multiple robots has no impact on the localization accuracy and that the actual localization accuracy for the set of parameters used is indeed lower than 0.3 m in all cases, which is close to the average error obtained in experimental testing as in Figure 6. 

**Figure 11:**
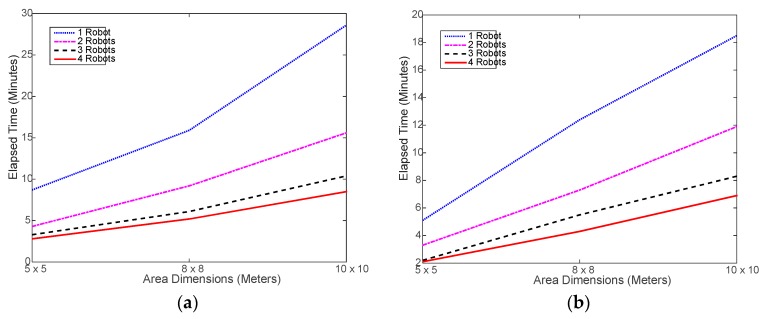
Simulated elapsed time of running the navigation and collecting required RSSI samples: (**a**) using distributed WiMAP algorithm; (**b**) using distributed DWiMAP.

**Figure 12:**
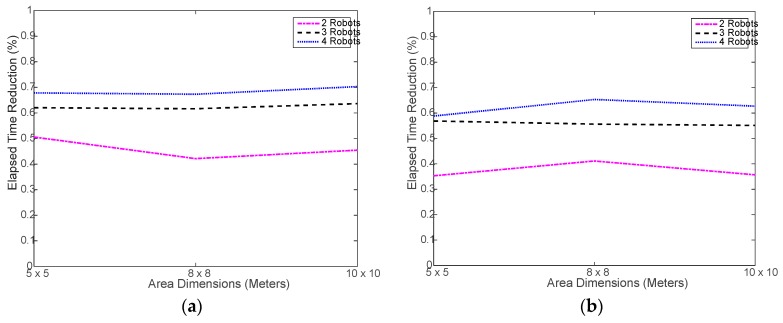
Simulated elapsed time reduction: (**a**) using distributed WiMAP algorithm; (**b**) using distributed DWiMAP.

Figure 11 shows a slight increase in the simulated run time compared to the real run time, shown in Figure 7. This is mostly caused by the overhead incurred by the real robot to slow down and, sometimes, stop before changing its direction and move again, which is not implemented in the simulation. Otherwise, the simulated navigation of the robot seems to be well implemented. On the other hand, Figure 12 shows that the less-overhead simulation environment allows for closer time reductions to the ideal limits than the real testing as shown in Figure 8.

**Figure 13:**
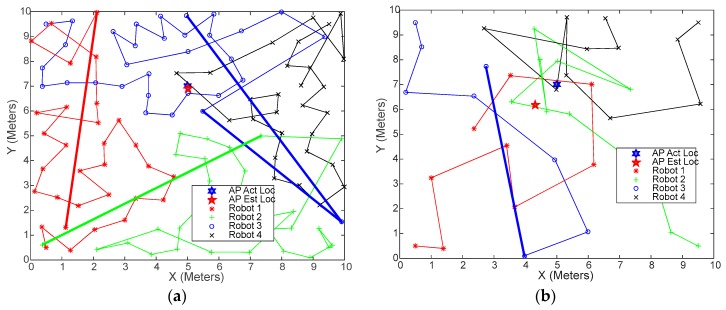
Robot navigation path showing the problem of the high overhead caused by the random search algorithm: (**a**) for distributed WiMAP algorithm; (**b**) for distributed DWiMAP.

As indicated earlier, during the experimental testing, individual robot’s elapsed times seemed to vary, and considerably sometimes. This problem was investigated using the simulation testbed and it was found that some robots’ navigation paths tend to get much longer toward the end of the random search process. Figure 13 shows sample navigation paths of four robots, each starting from one of the corners. The thick lines represent the routes taken by some robots to reach the last one or two sample locations. Note how long these routes are, compared to the average route length. The reason the robots are forced to do this is that the algorithm requires all cells in the environment to be fully covered. These “long shot” sample locations could be covered with much less effort and time, whether by the same robot or any other robot, had the search process been optimized.

**Figure 14:**
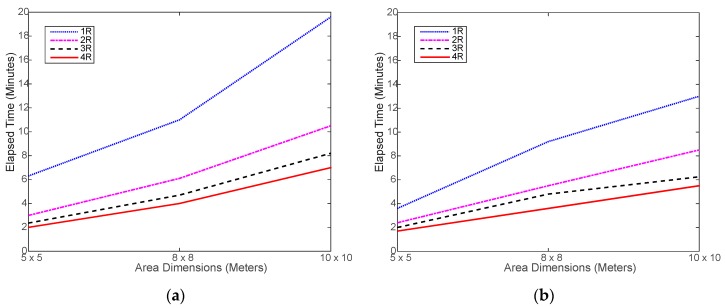
Enhanced simulated elapsed time after allowing the robot swarm to only cover 95% of the cells: (**a**) for distributed WiMAP algorithm; (**b**) for distributed DWiMAP.

A simple solution to this problem is to allow the robots to cover a certain minimum percentage of the cell, instead of all cells. To illustrate the idea, we reran the same scenario of Figure 11 while allowing the robot swarm to cover no less than 95% of the cells. The result was that the elapsed time dropped by almost 30% without affecting the localization accuracy, as shown in Figure 14. The reason is that the accuracy of WiMAP and DWiMAP are insensitive to the number of samples as long as they are sufficient. However, this solution may not suit all multi-robot applications. Therefore, the process of covering the cells needs be enhanced or optimized to allow the swarm to cover all cells with low cost.

As a final experiment, the performance of different access point localization algorithms was evaluated and compared in terms of localization error and execution time by the robot swarm. The algorithms are: the Centroid [15], Weighted Centroid [16], Particle Swarm Optimization [14], WiMAP [17], and DWiMAP [18] in a 25 m × 25 m with the access point at the center. Table 3 lists the results, which represent the average of 25 trials each, for one and four robots. The robots were used to randomly collect the RSSI samples, which were used as an input set to each algorithm. All algorithms, except DWiMAP, use uniformly distributed RSSI sample sets, whereas DWiMAP takes a selected small number of most effective sample locations. This allows DWiMAP to identify the location of the access much faster than the rest without sacrificing the localization accuracy. Even though both WiMAP and DWiMAP achieve almost the localization performance, which is the best among all algorithms, DWiMAP does that in one-fourth to one-third of the time.

## 4. Discussion

In the current approach, we used a collaborative mobile robot swarm to autonomously and efficiently detect the location of a wireless access point without any prior knowledge of the environment under investigation. The mobile robots are equipped with wireless communication devices that enable them to form a mobile ad hoc network for information exchange and status updates over a reliable communication channel. The robots are also equipped with onboard range and proximity sensors for path planning and obstacle avoidance and with accurate Odometry-based self-localizing capability.

Distributed versions of existing access point localization mechanisms were developed and implemented into the mobile robots in order to collaborate in identifying the location of the access point in an efficient, fast, and accurate manner.

The access point localization mechanisms were experimentally tested in obstructed indoor environments. In the experiments, the access point was placed randomly within the area of interest and the mobile robots were made aware of the dimensions of the area and their initial locations, in addition to the intended localization mechanism and the associated input parameters.

Experimental testing of the modified mechanisms demonstrated that the mobile robots were able to collaborate successfully to detect the location of the access point in nearly one-nth of the time needed by a single robot, where n is the number of active robots in the swarm, without affecting the localization accuracy or number of collected samples.

Because experimental testing is labor-intensive and time-consuming, especially in large-scale sites and in order to facilitate faster testing development of such mechanisms, a simulation testbed that imitates the real experimental testing was also developed and was verified in comparison with the experimental testing. The simulation testbed was used in identifying an excess overhead problem in the navigation algorithm and in mitigating its impact by more than 30%.

## 5. Conclusions

Collaborative mobile robot swarms have enormous potential in many useful indoor applications. A robot swarm can replace a team of human workers in performing well-defined collaborative tasks in hazardous environments such as nuclear reactors, industrial chemical processes, or minefields. It can also be used in optimizing the wireless coverage by running a fast periodical walk-through-testing for coverage holes or in tuning a fingerprinting based indoor wireless localization system in fast-changing dynamic environments such as factories, hospitals, and shopping malls.

This paper proposes one important application of a collaborative mobile robot swarm, which is identifying the location of an access point in a relatively large indoor obstructed environment. If the access point to be localized is illegitimate, causing security threats to the local network, then localizing and arresting it in a timely manner is of great importance.

Experimental testing, as well as simulation-based testing, demonstrated that using a collaborative mobile robot swarm, especially with a DWiMAP algorithm, can significantly reduce the time it takes to localize the access point, without degrading the localization accuracy of the localization mechanism used or increasing any other cost. Distributed versions of different state-of-the-art access point localization algorithms with a swarm of four robots consistently achieved between 70–75% reduction in the time needed to localize the access point, compared to one robot, without affecting the localization accuracy. In addition, DWiMAP achieved between 68–74% reduction in time, compared to all other algorithms, without affecting the localization accuracy. Finally, it is worth mentioning that using mobile robots to collect RSSI samples points provides more robust and accurate localization accuracy compared to humans. For example, in original WiMAP [17], the average reported localization accuracy of the experimental testing performed by humans was 1.36 m, whereas the average localization accuracy achieved by a mobile robot was between 0.2 and 0.3 m, which supports the argument that mobile robots can be more reliable and consistent than humans in collecting RSSI samples.

As a future work, the collaboration among the robots, in terms of task allocation and execution, needs to be optimized in order to minimize the overhead incurred by the collaboration effort. 

## 6. Patents

No patents have resulted from the work reported in this manuscript.

## Figures and Tables

**Table 1:** The ultrasonic sensor weights used in the experiments.

Ultrasonic Sensor	Left Motor Weight	Right Motor Weight
Left	70	−70
Front Left	100	−100
Front	150	−150
Front Right	−100	100
Right	−70	70

**Table 2:** The infrared sensor weights used in the experiments.

Infrared Sensor	Left Motor Weight	Right Motor Weight
Back Left	−20	20
Middle Left	20	−20
Front Side Left	40	−40
Front Left	60	−60
Front Right	−60	60
Front Side Right	−40	40
Middle Right	−20	20
Back Right	20	−20
Back	0	0

**Table 3:** A comparison of access point localization algorithms.

Algorithm	Localization Error		Execution Time		
	1 Robot	4 Robots	1 Robot	4 Robots	Reduction
Centroid [15]	2.30 m	2.60 m	226 min	56 min	75%
Weighted Centroid [16]	1.70 m	1.40 m	226 min	56 min	75%
PSO [14]	0.63 m	0.63 m	226 min	56 min	75%
WiMAP [17]	0.22 m	0.20 m	226 min	56 min	75%
DWiMAP [18]	0.19 m	0.19 m	59 min	18 min	70%
